# On the Affusion of Cold Water in Fever

**Published:** 1800-01

**Authors:** P. M. Martineau

**Affiliations:** Norwich


### On the Affufion of Cold Water in Fever, by Mr. Martineau, Surgeon, of Norwich.

---

### To the Editors of the Medical and Phyfical Journal.

Gentlemen,

I Should be extremely happy if I could draw attention to the practice of the *affufion of cold water* in the cure of fevers, which has been fo ftrongly recommended in a work of great merit, by Dr. Currie, of Liverpool; but which has not, fo far as I can learn, been followed in this part of the kingdom.

That ablution with cold water in fever fhould not have been more employed, is perhaps owing to the innovation it appears to make in the common mode of treatment; and that practitioners fhould remain fearful of cold in fevers, may arife in part

 from from the relief obtained from *sweating*, produced often by the opposite application of heat, forgetting that perspiration itself is the cooling process which nature sets up for carrying off preternatural heat.

In *external* inflammation, surgeons have not been timid in the application of cold, and experience there has confirmed the propriety of subduing inflammation, by taking off heat by cold applications. The analogy between external and internal diseases, attended with preternatural increase of heat, is great, and might lead to many pathological conclusions; but at present I shall content myself with giving a case or two, in which the *Affusion of Cold Water in Typhus* was attended with manifest advantage.

In November 1798, a young man, a farmer, about twenty years old, living four miles from Norwich, came to me, complaining of great lassitude, head-ach, loss of appetite, and costiveness; he had a quick, tremulous pulse; great dejection was marked in his countenance, and, in short, every appearance of Typhus. This was on a Thursday, and he had been complaining from the preceding Saturday. He was much fatigued with his ride, and it was with difficulty he returned home. I ordered him an emetic to be taken that evening, and a gentle dose of opening medicine for the following morning. I heard no more of him until the Saturday, when I was requested to go over to him. I found him at six that evening with every symptom growing worse, and his debility much increased. I prescribed a drachm of bark, to be given every two hours, and an opiate at bed time.

Sunday evening his pulse was 110, his tongue clear, skin hot and dry, his weakness greater. He had taken the bark very regularly, which I desired might be continued, as well as his opiate at bed time.

Monday, at six in the evening, pulse as yesterday, heat pungent, head-ach with wandering, but not absolutely delirious, his strength less. The bark had purged him, notwithstanding laudanum had been given twice, beside the night draught.

In this state, with the bark purging, and the disease making an alarming progress, I determined, although I was unable to measure his heat, and too far from home to wait for a thermometer, to make trial of the Affusion of Cold Water. My patient was taken out of bed, and while he was supported, standing naked in a tub, I poured the largest hand-bason of pump water all over him. The shock was considerable to *him*, and the father and mother, who were present, thought, I believe, I should be the death of their son. He was wiped dry, and immediately returned to bed—his pulse then beat only 70—he was was cool, and ſaid he had not felt himſelf ſo comfortable, and particularly in his head, for many days. Much pleaſed with this effect, but uncertain whether it would laſt, I went down ſtairs, and waited an hour; on my return to him, his pulſe had not quickened, nor had the heat returned. I left orders to repeat the cold water, if he became hot during the night, but there was no occaſion for it; he ſlept well, and had a gentle perſpiration; and although I daily intended repeating the affuſion, had the heat returned, I never found it neceſſary. His ſymptoms ſeemed at once arreſted, but continued in a ſlight degree until the 14th day, when his appetite and natural ſleep returned, and he ſoon after recovered his ſtrength and health.

I ſhould mention, that from the evening in which the affuſion was uſed, I only ordered two or three ſmaller doſes of bark in a day, conjoined with a few drops of laudanum to check the purging, and 24 drops at night, until the 14th day, when all medicine was laid aſide. The bark, in ſuch a ſmall quantity, can ſcarcely be ſuppoſed to have contributed to the recovery of this patient; and I will add, that in the largeſt quantity, I never ſaw it of ſervice, either in ſtopping of Typhus, or moderating the ſymptoms, unleſs given in the firſt two or three days, when I know it will often put a ſtop to the diſeaſe; but it muſt be given with the ſame aſſiduity as is required to check the return of a true intermittent. It is the time of giving, as well as quantity of bark, which muſt render it ſuccesſful in Typhus.

Dr. Currie in his admirable work mentions, that he finds the greateſt benefit from affuſion, when uſed in the firſt days of fever; and this I believe, for the very reaſon which makes the bark, and ſome other remedies, chiefly uſeful in the commencement, viz. that if the diſeaſe has had time to obtain its true character, or, in the language of Dr. Darwin, "the morbid "febrile catenation is ſtrongly formed," it will go on its own duration, in ſpite of our efforts to ſtop its natural termination. In my patient, however, the affuſion was not tried till the ninth day, ſtill the ſudden impreſſion made by it was ſo powerful as to produce ſuch a mitigation of every ſymptom as to leave no farther apprehenſion for his ſafety, although no poſitive criſis came on before the fourteenth day, when the appetite and ſleep marked the concluſion.

In January, 1799, the Lincolnſhire militia were quartered in this city; their barracks were terribly crowded, ill ventilated, below the ſurface of the ground, and damp; the weather was extremely cold, and the men, after parade, frequently complained of having caught cold. After a ſhort time the diſeaſe put on a mixed character of Typhus and Peripneumonia, the

Perip-

Peripneumonia putrida of Sauvages, and many died. I was requeſted at this time, by the Colonel, Lord Buckinghamſhire, and Mr. Cooper, the ſurgeon of the regiment, who had been indefatigable in his attention to the men, to viſit the hoſpital, which was a ſmall houſe, in which were thirty, in all the ſtages from an alarming commencement to a fatal concluſion. Two were brought in the evening, while I was there, who had been ill a *few days* only; and as there was conſiderable heat on the ſkin, I recommended the affuſion, which was immediately complied with. The pulmonic ſymptoms might have been conſidered an objection to the trial, but the fatality of the diſeaſe led me to adopt a practice, which at firſt I ſhould not have had courage to have employed. In theſe caſes no very *immediate* relief was given, but both the men recovered with leſs ſevere ſymptoms than moſt of their comrades; bliſters, however, were applied to them, and had not been uſed in the other caſes. I have mentioned theſe two caſes, to ſhew that even with pneumonic affection there aroſe no *inconvenience* from the application of cold water. An immediate ſtop was put to the contagion by the men being, the day after my viſit, ſent out of the barrack to ſeparate houſes.

In March laſt, Mr. Reeve, a pupil of mine, and whoſe attention and accuracy may be depended on, viſited a poor boy, ten years old, who was in the fifth day of a Typhus, four of which he had been confined to his bed. He at firſt gave him an emetic, and ſome bark; but not finding him better the following evening, he applied the affuſion of cold water, during the hot ſtage of the evening exacerbation. The pulſe immediately fell from 120 to 98, the head-ach and heat were greatly diminiſhed, and ſome ſleep and a gentle perſpiration followed. The affuſion was uſed the next day at noon, and again at night, with the ſame advantage, and once more the following evening. On the 9th day, the fever terminated, and the boy rapidly recovered, having taken no medicine after the application of the water.

A fortnight afterwards, a brother of the above, aged eight years, was ſeized with the ſame fever; the affuſion was applied on the *second* day with the greateſt advantage, as he had no return of fever for four days, when ſome cold winds blowing upon him in bed, produced a relapſe; the ſymptoms were more violent, and delirium and coma were added. On the evening of the ſecond day, of the ſecond attack, when the heat was very conſiderable, cold water was thrown all over him, and with an aſtoniſhing good effect, for he had no return from that time, and ſoon recovered, without having taken any medicine during the whole period of the diſeaſe.

On

On the 8th of May, the ſiſter of the two boys, a girl of ſix years, was attacked with Typhus, but with ſymptoms leſs violent. The affuſion was applied twice at the commencement of the fever, and ſhe ſoon recovered without any medicine.

The above caſes may, I hope, be an additional inducement to the practice they are intended to eſtabliſh; many circumſtances ought to be taken into conſideration before its general application; and I cannot do better than refer ſuch of your readers who have not already peruſed Dr. Currie's work, to the book itſelf, and conclude in his words—" Affuſion may be " ſafely uſed at any time of the day, *when there is no ſenſe of* " *chillineſs preſent, when the heat of the ſurface is ſteadily above* " *what is natural, and when there is no general or profuſe per-* " *ſpiration.*"

I am, Gentlemen,

Your obedient humble ſervant,

*Norwich, Dec.* 13, 1799. P. M. MARTINEAU.

